# Canine Circovirus in Foxes from Northern Italy: Where Did It All Begin?

**DOI:** 10.3390/pathogens10081002

**Published:** 2021-08-09

**Authors:** Giovanni Franzo, Maria Luisa Menandro, Claudia Maria Tucciarone, Giacomo Barbierato, Lorenzo Crovato, Alessandra Mondin, Martina Libanora, Federica Obber, Riccardo Orusa, Serena Robetto, Carlo Citterio, Laura Grassi

**Affiliations:** 1 Department of Animal Medicine, Production and Health (MAPS), University of Padua, 35020 Legnaro, Italy; marialuisa.menandro@unipd.it (M.L.M.); claudiamaria.tucciarone@unipd.it (C.M.T.); giacomo.barbierato@studenti.unipd.it (G.B.); lorenzo.crovato@studenti.unipd.it (L.C.); alessandra.mondin@unipd.it (A.M.); laura.grassi.2@phd.unipd.it (L.G.); 2 O.U. of Ecopathology, SCT2 Belluno, Istituto Zooprofilattico Sperimentale delle Venezie (IZSVe), 32100 Belluno, Italy; martina.libanora@studenti.unipd.it (M.L.); fobber@izsvenezie.it (F.O.); ccitterio@izsvenezie.it (C.C.); 3 S.C. Valle d.’Aosta—National Reference Centre Wildlife Diseases, Istituto Zooprofilattico Sperimentale del Piemonte, Liguria e Valle d’Aosta (IZS PLV)—Ce.R.M.A.S., 11020 Quart, AO, Italy; riccardo.orusa@izsto.it (R.O.); serena.robetto@izsto.it (S.R.)

**Keywords:** CanineCV, fox, evolution, Italy, history, phylogenesis

## Abstract

Canine circovirus (CanineCV) is a recently identified virus affecting both domestic and wild carnivores, including foxes, sometimes in presence of severe clinical signs. Its circulation in wild animals can thus represent a potential threat for endangered species conservation and an infection source for dogs. Nevertheless, no data were available on its circulation in the Alps region of Northern Italy. In the present study, samples collected from 186 foxes in the period 2009–2020 from Valle d’Aosta and Veneto regions were tested using a real-time PCR assay, demonstrating a viral circulation of approximatively 2–5%, depending on the considered regions. Two complete or almost complete genome sequences were obtained, highlighting that the detected strains were part of a so defined “fox only” clade, which suggests that, despite common contact opportunities, Alps foxes are not involved in frequent transmission events to domestic dogs. Such genetic isolation could be at least partially attributed to some sort of independent evolution occurred in the foxes, leading to species barrier. Additionally, CanineCV strains in foxes from Italy were unexpectedly related to those previously identified in foxes from the United Kingdom and Scandinavian area. Combining the history of fox distribution in Europe since the last glacial maximum (LGM) with the viral history allowed us to speculate a long-standing coexistence between European canine circovirus and this host, justifying the peculiar geographic distribution and evolutionary paths of the fox infecting clade.

## 1. Introduction

The genus *Circovirus* includes small, non-enveloped, icosahedral viruses with a single-stranded circular DNA (ssDNA) genome of approximately 2 kb in size, featured by the presence of two main open reading frames (ORFs) oriented in opposite directions. ORF1 encodes for the Rep proteins involved in viral replication, while ORF2 encodes for the Cap protein, the only constituent of the viral capsid [1].

Like other ssDNA viruses, they display a high mutation rate, which can lead to the genesis of remarkable genetic variability, allowing them to readily adapt to new environments [2]. Although traditionally considered host-specific, different studies, especially dealing with porcine circoviruses, highlighted a certain plasticity in terms of host tropism [3,4,5]. Therefore, the role of host jump in the origin of new circoviruses has been speculated [6]. Recently, many circoviruses have been described from several hosts by different methods [1]. Among these, canine circovirus (CanineCV), identified in dogs with vasculitis and/or hemorrhagic gastroenteritis in the United States in 2012 [7], has been reported in several countries over time, including USA, Italy, Germany, and Taiwan, in presence of different clinical signs such as vomiting and diarrhea with hematochezia, necrotizing vasculitis and granulomatous lymphadenitis [7,8,9,10,11,12]. CanineCV has attracted a certain interest because of the potential clinical relevance in dogs, since some pieces of evidence suggest its potential role in disease occurrence, both as a direct cause or co-factor. Nevertheless, a consistent demonstration of its pathogenic role is still lacking [12]. Further studies have demonstrated its circulation in wild members of the genus *Canis*, including wolves (*Canis lupus*), but also in foxes (*Vulpes vulpes*) [13,14] and badgers (*Meles meles*) [15]. Interestingly, in foxes, CanineCV has been identified in subjects affected by meningoencephalitis, with its nucleic acid localized in histological lesions of the cerebrum by in situ hybridization [14]. Fox-related strains resulted relatively genetically distant from those identified in other hosts [16], to the point that they have been considered as an independent species (referred to as *Fox circovirus*) for a long time.

In an evolutionary perspective, the role of wild animals as a source for domestic animal infection has been proposed for several viral diseases. For example, canine parvovirus (CPV), which shares several biological features (e.g., ssDNA genome, dependency from host polymerase for replication, high evolutionary rate, target cells, etc.) with CanineCV, is assumed to have originated from feline parvovirus (FPV) or FPV-like virus adaptation to the canine host through wild intermediates, potentially red foxes [17,18]. However, it remains to be established if a similar path occurred also for CanineCV.

In Italy, red foxes (*Vulpes vulpes*) are the most present wild carnivore. Their geographical distribution encompasses several ecological environments and habitats, and their population size enables this species to play the role of epidemiological reservoir for many agents that can infect all susceptible species sharing the same habitats [19]. Moreover, the last decades have been featured by abandonment of human activities, as agriculture and farming, in many marginal areas, followed by a return of a certain extent of wilderness. In such a context, many wild species, characterized by ecological plasticity, have increased their consistency. Among these, the red fox population is easily exploiting the resource availability in an environment with scarce or no disturbance [20]. Foxes are frequently sighted in urban and peri-urban localities, becoming a sort of connection between the wild and the anthropic environments. At the same time, the last decades have featured a return to extensive agro-pastoral activities, involving the presence of sheepdogs, and nature-oriented recreational activities [20,21,22]. Finally, an evident increase in population size, migration, and introduction from foreign countries have been observed for other European wild carnivores, such as the wolf (*Canis lupus*) and the golden jackal *(Canis aureus),* some of which were proven to be susceptible to CanineCV infection and thus possibly contributing to its epidemiology [15].

The present study aims to investigate the frequency and distribution of CanineCV in foxes from two alpine Italian areas, namely the Valle d’Aosta region in the Italian Western Alps (IWA), bordering France and Switzerland, and the Belluno province of Veneto region in the Italian Eastern Alps (IEA), bordering Austria and near to Slovenia. The fox population of these areas represents a continuum with that of the bordering countries [23]. 

## 2. Results

### 2.1. CanineCV Diagnosis and Genetic Characterization

**Figure 1:**
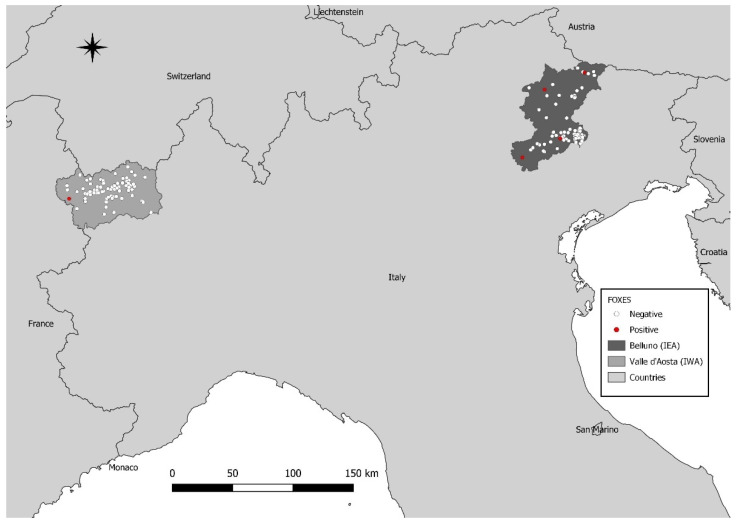
Map of sampling location sites. The included regions have been highlighted with different grey shades. CanineCV negative and positive samples are reported in white and red, respectively.

One out of 70 foxes (1.4%), collected from IWA in 2009 (FoxVa_61) and 5 out of 115 (4.3%) sampled in IEA (FoxVe_44, FoxVe_66, FoxVe_67, FoxVe_68, and FoxVe_95) between 2017 and 2018, tested CanineCV positive (Figure 1).

For none of the CanineCV positive animals, clinical signs nor lesion ascribable to infectious diseases were observed. Viral titers were constantly low (Table 1) and only one complete (FoxVa_61) (Acc. Number MZ407653) and one partial (FoxVe_66) (1051nt) genome sequence were obtained. 

Sequence analysis of the complete FoxVa_61 genome revealed the typical genome organization of CanineCV, with a 2063 nucleotide long genome encoding a 912 (303aa) and 897 (298aa) ORF1 and ORF2 genes. 

### 2.2. Phylogenetic Analysis

**Figure 2:**
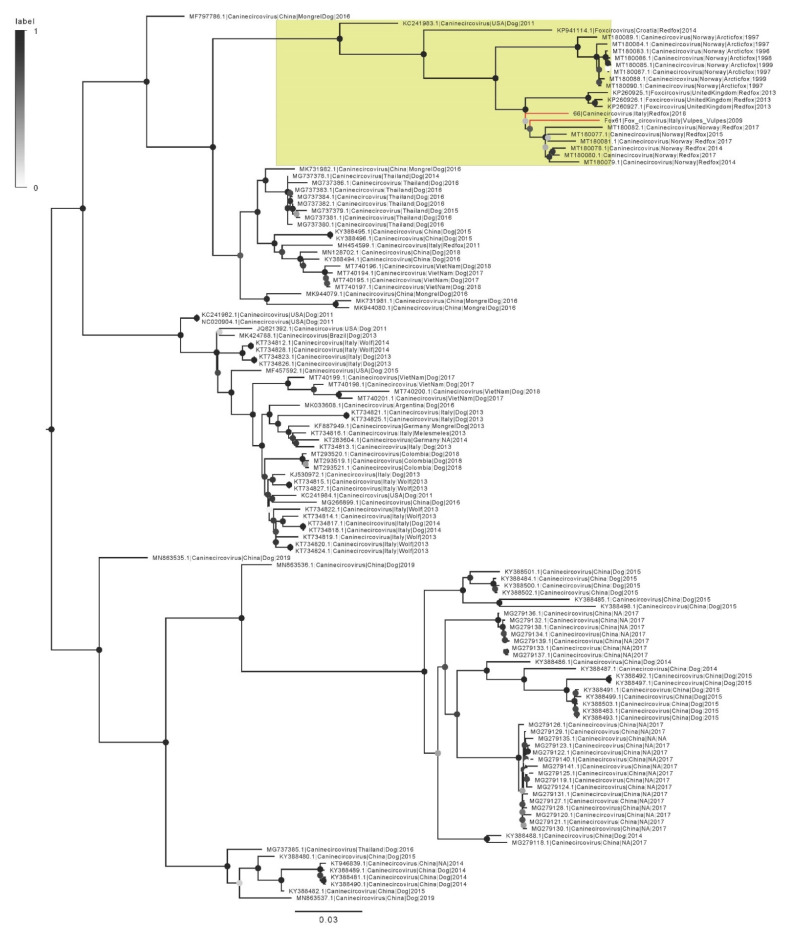
Midpoint-rooted maximum likelihood phylogenetic tree of CanineCV. Branches corresponding to the sequences obtained in the present study have been colored in red while the clade including only fox sampled viruses has been highlighted in yellow. The bootstrap support of each node has been color-coded from white (lower values) to black (higher values).

Sequence FoxVa_61 was part of a cluster including strains collected only from foxes and it was closely related to CanineCV sequences sampled in Norway, and to a lesser extent to UK sequences (Figure 2) (overall, the genetic distance compared to other CanineCV part of the “fox-only” cluster ranged between 4.2% and 11.5%). 

Similarly, FoxVe_66 was also part of the previously mentioned “fox cluster” (showing a genetic distance compared to other CanineCV part of this cluster ranging between 3% and 9%), although quite distantly related to FoxVa_61 (p-distance = 0.055). Both sequences were distantly related to other CanineCV sequences, including those collected from Italian domestic dogs, wolves, and foxes (Figure 2).

### 2.3. Phylodynamic Analysis

No evidence of recombination among sequences included in the study was detected.

Based only on sampling date, tMRCA ancestor was estimated 163.36 years (ya) [95HPD: 118.58–215.45], while the corresponding evolutionary rate was 1.21 × 10^−3^ [95HPD:8.80 × 10^−4^–1.56 × 10^−3^] substitution/site/year. Population dynamics estimation revealed an essentially constant population size until approximately 2000, when a significant rise was observed, followed by a similarly marked decline after 2010.

The separation events between canine and fox clade, based on other circovirus speciation estimations, was predicted to have occurred 10.56 millions of year ago (mya) [95HPD: 7.22–18.56].

When the internal calibration node based on last glacial maximum (LGM) was used, tMRCA was remarkably backdated, since it was estimated 56615.81 ya [95HPD: 37288.85-76060.77]. The evolutionary rate was 3.75 × 10^−6^ [95HPD: 2.39 × 10^−6^–5.31 × 10^−6^] (Figure S1 and Table S1). 

## 3. Discussion

The present study results extend and update CanineCV epidemiology of foxes in Italy, revealing an infection frequency between ~2 and 5% depending on the considered areas, in line with or higher than other studies performed in the same country. Particularly, no evidence of fox infection was reported in central Italy by Zaccaria et al., (2010) [15], while de Arcangeli et al., (2020) [13] reported a 3% detection frequency from fox samples in Tuscany. Although circovirus infection is often persistent, the likelihood of detecting an active viremia or shedding can be considered low. None of the positive foxes displayed clinical signs or lesions clearly ascribable to infectious disease, which poses in favor of subclinical infection or recovered subjects. Due to the sampling nature and purpose, an accurate diagnostic approach was challenging. However, clinical signs, if present, were reported by hunters. Thereafter, a standard necropsy was performed on each animal and specific diseases were investigated based on the observed findings. Therefore, severe to moderate syndromes should have likely been identified, even though more subtle diseases could have passed undetected. Accordingly, almost all positive animals showed extremely low viral titers, suggestive of a previous infection tail rather than active infection. Therefore, even apparently rare detections can be considered of relevance. Unfortunately, the need of dealing with convenience and archive samples impedes the evaluation of the real prevalence. Despite these limitations, significant data could be obtained from the Alpine regions, which are of particular interest since a relevant number of foxes live in this area. Additionally, the Alps represent at the same time a physical barrier and the main “point of contact” between Italy and other countries, at least from a wild animal perspective. Particularly, the Valle d’Aosta region neighbors Switzerland and France, while Belluno province shares a limited border with Austria and is part of a continuum mountain area with Friuli Venezia Giulia region, which in turn neighbors the Balkans and Austrian regions. Of note, all previously reported Italian CanineCV strains were part of a different cluster, which includes variants collected from domestic dogs and other wild animals, but not foxes, except for only one sequence (MH454599.1) [13,15]. Based on these evidences, the interaction between foxes and domestic dogs seems not relevant in viral strain exchange and maintenance, at least in the considered areas. Why alpine populations demonstrate such a peculiar host/virus combination remains to be elucidated. Unfortunately, the extremely limited data availability, ascribable to the few performed studies and the challenges in obtaining high-quality sequences from low viremic individuals, currently prevents definitive conclusions. Particularly, no investigation was performed to evaluate the viral circulation in dogs living in the considered areas and further dedicated studies would be of interest to confirm the suggested hypothesis and characterize CanineCV strain exchange between wild and domestic species. However, such an exchange, if present, would mainly involve wandering owned dogs, hunting or shepherd dogs, since properly “stray dogs” are extremely rare or absent in IWA or IEA.

Reconstruction of the history of CanineCV is a challenging task. The evidence that the detected strains are part of a clade including fox-only viruses suggests a long-term circulation in a close population. If this is due to limited contacts with another canine/carnivore populations or host-specific adaptation remains to be confidently established. Nevertheless, the interaction with other carnivores proven susceptible to CanineCV infection is likely, since several of these species share the same habitat with the investigated fox populations [15,20] and circoviruses typically demonstrate a high environmental resistance, thus facilitating indirect transmission. Moreover, frequent contacts with domestic dogs can be expected both because of dog encroachment in the wild environment for hunting, pasture, and recreational purposes and fox intrusion in urban or peri-urban areas in search of food. Therefore, viral adaptation leading to some sort of genetic barrier could be more likely. Divergence dating based on estimated speciation events featuring other circoviruses suggested an extremely ancient separation of fox and canine circoviruses, in agreement with what reported by Das et al., [24]. Such ancient divergence must be considered with caution since inevitably affected by the uncertainness in the original calibration. Additionally, the rapid adaptation to the host after a more recent host jump could have affected the evolutionary rate and biased the estimations. Nevertheless, a quite ancient separation between the two viral groups can be stated. Such an ancient origin, combined with the strong strain clustering of fox viruses and the high genetic diversity, might challenge the idea of canine and fox circovirus being part of the same species, although supported by the formal species demarcation criteria defined by ICVT. 

Such independent evolution was mirrored by viral circulation within the host populations. Currently, only European sequences are available.

Therefore, viral dispersal patterns must be considered in the context of fox movements within Europe. Surprisingly, Italian strains display a closer relationship with the ones collected from Norway and UK, than with other Croatian and even Italian viruses. 

Based on this scenario, two conflicting hypotheses can be advocated. The first one involves the recent contact between distant populations. Fox dispersal can be extremely variable, being some foxes sedentary, while others migrate for hundreds of kilometers, facilitating viral dispersal [25]. Genetic analysis of the fox population in Italy highlighted the presence of at least two quite separate animal clusters, one including foxes from the far east Italy, which are related to Austrian, Slovenian, and Croatian individuals, and the other composed of Italian foxes from Veneto and Trentino Alto Adige regions [23]. This separation is likely due to the presence of geographical barriers impeding animal dispersal and limiting gene flows in foxes. This structure was also shown as relevant in conditioning the spreading of rabies and distemper virus in Italian foxes [23]. Such evidence could be extended to explain the relevant genetic distance between the two Italian CanineCV strains from the Alps. However, although relatively rare, exceptions to this rule were observed, being foxes of different clusters admixed. Comparably, rabies introduced in far eastern regions was able to cross the eastern barriers and penetrate the central areas. Similarly, distemper, first notified in Bolzano province (Trentino-Alto Adige/Südtirol), was able to cross western barriers [23]. Nevertheless, whether such fox spreading dynamics can explain the close relationship between Southern and Northern Europe CanineCV strains is hard to establish.

An alternative hypothesis could involve a far more ancient origin of CanineCV distribution, determined by historical European fox migrations. During the last glacial maximum (LGM), approximatively 26 thousand years ago, several species were pushed southward [26]. Fossil evidence suggests that for more than seven thousand years (kya) (23–16 kya) fox distribution was limited to the Iberian Peninsula, Italy, the Balkans, and certain regions of France [27]. Thereafter, a northern expansion began, reaching the current range by the mid-Holocene (8.2–4.2 kya) [26]. Mitochondrial and nuclear gene analysis indicates that Italian foxes contributed significantly to central European populations, which in turn colonized Norway via Denmark across a land bridge to Sweden [28]. 

It could be speculated that viral dispersal followed the fox migration pattern. Such a scenario would however be incompatible with (1) the estimated tMRCA of CanineCV (i.e., some decades/centuries ago considering the higher bound estimate), (2) the circovirus (and ssDNA virus in general) evolutionary rate.

Nevertheless, it must be kept in mind that the “time-dependent rate phenomenon” (TDRP) has been largely recognized in viruses [29,30]. Additionally, paleovirological analyses have shown that many ancient endogenous viruses related to RNA and ssDNA viruses exhibit high similarity to their modern-day counterparts despite being millions of years old, and such evidence has been provided for circovirus also [31,32,33]. It is becoming increasingly clear that viral evolutionary rate estimates are systematically negatively correlated with the time scale of rate estimation, continuously decreasing as the measurement time scale increases [34]. Fully comparable results were obtained in the present study when the molecular clock was calibrated based on ancient geological events rather than tip-dating (Figure S1). Although a complete discussion of the topic is out of the scope of the present manuscript, different factors have been hypothesized to determine TDRP. Deleterious mutations can persist within viral populations for significant amounts of time before being purged, causing rates estimated over short timescales to be overestimated. On the contrary, long-term evolutionary rates are the product of coevolution between viruses and their slowly evolving hosts, and many constraints are in place for proper biological functions and host interaction, leading to site saturation and convergent evolution, which in turn causes an underestimation of the evolutionary rate. Moreover, limitations of the currently available biostatistical tools and models are also contributing to the observed phenomenon. A more detailed review of the TDRP causes can be found in Ho et al., 2011 [29].

Based on these considerations, a long term CanineCV-fox pattern dispersal and co-evolution cannot be excluded. Interestingly, the estimation of ancestor date of “fox circovirus” mirrors the reconstruction of fox migration across Europe based on fossil evidence. Despite the broad selected *priori*, tMRCA of this clade was predicted in 17 kya, during the LGM, when the fox area was constrained at most [26,27]. Even more surprisingly, the origin of the strains detected in UK was dated approximatively 8 kya, which agrees with the current knowledge about the separation of British and Irish foxes from continental Europe (5.7e 14.5 kya) and is in keeping with the last overland connection between Britain and continental Europe, via Doggerland, which existed in the Holocene, and finally flooded around 7.8 kya [35]. Therefore, although not conclusive, the congruence between the estimation of fox and viral migrations across Europe are at least suggestive of our hypothesis plausibility. 

A combination of the two scenarios could be true, being the currently observed pattern the result of both ancient migration events dispersing an initial viral “nucleus” across Europe, and more recent, sporadic spread associated with the fox population mixing.

The discordance compared to the fox circovirus estimated using other circovirus species divergence, might seem contradictory. However, several factors can explain the different analysis results. In addition to the previously mentioned unavoidable uncertainness associated with the molecular clock calibration based on the estimated speciation events, it must be remembered that the sequenced “fox circovirus” strains are just a small subset of the whole population (i.e., a limited sample of the strains circulating in Europe). Because of the migration events, founder effects and reduction of host population size during LGM, several bottlenecks can have occurred. Therefore, the tMRCA estimated using geological events and available European sequences does not probably reflect the ancestor of all “fox circoviruses” but rather the ancestor of the currently circulating European viruses, whose origin is orders of magnitude more recent than the original “speciation” event.

Both technical limitations and, especially, the low sequence number of fox-derived sequences, originating from just a few countries, prevent any definitive deduction and further studies will be necessary when more data, theoretical background and analysis tools will become available. 

## 4. Materials and Methods

### 4.1. Samples

With the aim of evaluating the presence of CanineCV in red foxes from northern Italy, archive samples were selected and analyzed. Particularly, pools of organs originated from 70 foxes found dead or shot during the regular hunting season 2009/2010 in Valle d’Aosta region (IWA) were included in the study. Animal signalment and sampling location are reported in Table S2. After culling, carcasses were delivered to Centro di Referenza Nazionale per le Malattie degli Animali Selvatici (Ce.R.M.A.S.), Istituto Zooprofilattico Sperimentale Piemonte, Liguria e Valle d’Aosta, where necropsies and routinary diagnostics were performed. Similarly, 115 spleen samples collected between 2017 and 2020 from foxes regularly hunted or found dead in the Belluno Mountains (IEA) were provided, according to a material transfer agreement, by the Istituto Zooprofilattico Sperimentale delle Venezie (IZSVe). Sample metadata are provided in Table S2. All samples were delivered to the laboratory of veterinary infectious diseases of the Dept. of Animal Medicine, Production and Health (MAPS), Padua University, and stored at −80 °C until processing.

### 4.2. CanineCV Diagnosis and Sequencing

DNA was extracted from 200 µL of organ pools or spleen homogenate using the DNeasy Blood and Tissue Kit (Qiagen, Hilden, Germania), according to the manufacturer’s instructions. CanineCV diagnosis was performed on available samples using a previously described real-time PCR [13].

Thereafter, whole genome sequencing was attempted on positive samples using classical PCRs followed by Sanger sequencing. For this purpose, several overlapping PCRs were designed (Table 2). 

The classical PCR reactions were performed using the Platinum™Taq DNA Polymerase kit as follows: 5 µL of extracted DNA were added to a standard mix composed of 1X PCR Buffer, 1.5 mM MgCl_2_, 0.2 µM of each dNTP, 0.5 µM of each primer and 2 U of Platinum™Taq DNA Polymerase. Molecular biology grade water was added up to the final volume of 25 µL. The following thermal protocol was selected: 94 °C for 2 min, followed by 45 cycles at 94 °C for 30 s, 50 °C for 30 s, and 72 °C for 50 s. A final extension phase at 72 °C for 5 min was also performed. The presence and specificity of PCR products were assessed by 2% SYBR^TM^ Safe (Invitrogen, Waltham, MA, USA) stained agarose gel electrophoresis. Positive samples were Sanger sequenced at Macrogen Europe (Amsterdam, The Netherlands) in both strands using the same PCR primers. Chromatograms were evaluated with FinchTV (http://www.geospiza.com, accessed on 15 June 2021) (Seattle, Washington, DC, USA) and consensus sequences were obtained using CromasPro (Version 2.0.0, Technelysium Pty Ltd., South Brisbane, QLD, Australia).

### 4.3. Phylogenetic Analysis

All available CanineCV complete or nearly complete genomes were downloaded from GenBank (Accessed on 14 April 2021). Only sequences with available collection dates were included in the study. Reference sequences were aligned with those obtained in the present study using MAFFT [36]. Recombination occurrence was tested using RDP4 [37]. The primary scan was performed using RDP, GENECONV, Chimaera, and 3Seq, while recombination was confirmed with the whole set of available methods. Method settings were adjusted based on the dataset features according to the RDP4 manual. Recombination events were accepted only if detected by more than two methods with a significance level of *p* < 0.001 with Bonferroni correction. The presence of an adequate phylogenetic signal was assessed through likelihood mapping and phylogenetic analysis was performed using IqTree [38], selecting as substitution model the one with the lowest Akaike information criterion (AIC), calculated with a JmodelTest [39]. The clade reliability was assessed by performing 10,000 ultrarapid bootstrap replicates.

### 4.4. Phylodynamic Analysis

CanineCV origin and evolution were estimated using the serial coalescent-based approach implemented in BEAST 1.10 [40], implementing a Bayesian framework allowing to contextually estimate viral population features, including the time to most recent common ancestor (tMRCA), evolutionary rate and population size, accounting for phylogenetic and parameters uncertainness.

The best substitution model (GTR+G+I) was selected based on the Bayesian information criterion, calculated using Jmodeltest [39], while the molecular clock and population dynamic model were selected based on marginal likelihood calculation and comparison using the path sampling and stepping stone method (Table S2) [41].

The final estimations were obtained by performing a 200 million generation Markov chain Monte Carlo run, sampling parameters and trees every twenty thousand generations. Results were visually inspected using Tracer 1.5 and accepted only if mixing and convergence were adequate and the estimated sample size was greater than 200 for all parameters.

Parameter estimation was summarized in terms of mean and 95% highest posterior density (HPD) after the exclusion of a burn-in equal to 20% of the run length. Maximum clade credibility (MCC) trees were constructed and annotated using Treeannotator (BEAST package).

Two additional analyses were performed on the same dataset by calibrating the molecular clock using a dated geological event, namely the restriction and following expansion of the European fox population due to the last glacial maximum (LGM) [26] and the estimated speciation of other circoviruses (namely porcine circoviruses 1 and 2, and swan, goose and duck circovirus) [24]. To this purpose, in the first analysis, the node leading to the monophyletic clade including only fox circoviruses was attributed an *a priori* origin with normal distribution having mean 20 kya, and standard deviation of 3000 yrs. For consistency, the same substitution model (GTR+G), clock model (random local clock), and population dynamics (non-parametric skyline model) of the previous analysis were maintained. In the second one, the two-calibration node were attributed a broad *a priori* normal distribution with mean 10 ± 7 mya and 20 ± 10 mya, respectively. Analyses were performed as previously described.

## Figures and Tables

**Table 1:** Viral titer detected in CanineCV infected foxes. Available metadata are also reported.

Scheme.	Municipality	Age Category	Collection Date	Origin	Viral Titer (copies/mL)
FoxVa_61	La Thuile (AO)	Adult	2009/2010	Shot down	8.94 × 10^4^
FoxVe_44	Comelico Superiore (BL)	Adult	06/02/2017	Found dead	6.22
FoxVe_66	Colle Santa Lucia (BL)	Adult	08/10/2018	-	3.14 × 10^4^
FoxVe_67	Belluno	Adult	04/01/2018	Shot down	22.4
FoxVe_68	Belluno	Adult	04/01/2018	Shot down	1.96
FoxVe_95	Fonzaso (BL)	Adult	14/01/2018	Shot down	1.04 × 10^1^

**Table 2:** List of PCR primers used in this study. The primer position is based on the reference sequence NC_020904.

Name	Sequence (5′-3′)	Position	Amplicon Size	PCR
CanineCV-822-841-F	CTATCGGCGGTTGACCTCTA	822–841	389	1
CanineCV-1193-1212-R	CGACACTTCAACATCCCAGA	1193–1212		
CanineCV-1020-1040-F	CGTTTACCTGTTCACCCCCCT	1020–1040	1974	2
CanineCV-909-931-R	AGCGAGAGGCCTTTATCTTTCAG	909–931		
CanineCV-1F	GCAGTCGCAGATGAAACAGT	1817–1836	401	3
CanineCV-1R	TCCCGGCCACAGATTAAGTA	136–155		
CanineCV-2F	CGAGGCTTGCGAGAGCTG	451–468	651	4
CanineCV-2R	AAACGCACTTCAGTGTCACG	1083–1102		
CanineCV-3F	GAGGGCGTTTACCTGTTCAC	1015–1034	542	
CanineCV-3R	TCTTGACGGGGAAGATCAAG	1538–1557		
CanineCV-4F	GGTGGCTCCAATCTTCCTG	1474–1492	573	5
CanineCV-4R	TGTGCTGTGTCTGTGACGAG	2028–2047		

## Data Availability

All relevant data are provided in the present manuscript. Obtained sequences have been submitted to GenBank.

## Supplementary Materials

The following are available online at https://www.mdpi.com/article/10.3390/pathogens10081002/s1. Table S1: Summary of canineCV population parameters estimated using different combinations of population dynamics, molecular clock calibration, and type. Time to the most recent common ancestor (in years), evolutionary rates (substitution/site/year) and marginal likelihood estimation results (estimated using path sampling (PS) and stepping stone (SS) methods) are reported; Table S2: summary table of metadata available for the samples included in the present study; Figure S1: maximum clade credibility tree of CanineCV, calibrated using a dated geological event (i.e., the restriction and following expansion of the European fox population due to the last glacial maximum). For graphical reasons, the “fox-only” cluster is displayed while the rest of the tree has been collapsed. The estimated ancestral strain age is reported nearby the corresponding node, while the posterior probability is depicted as a color-coded circle.
