# Supplementary material for: Canine Circovirus in Foxes from Northern Italy: Where Did It All Begin?
Source: Pathogens. 2021 Aug 9;10(8):1002. doi: 10.3390/pathogens10081002 (PMC8400258; doi:10.3390/pathogens10081002)
Supplement: Supplementary file 1 [file pathogens-10-01002-s001.zip › supplementary Table S1 and Table S2.pdf]

pathogens

MDPI

Table S1. Summary of canineCV population parameters estimated using different combinations of population dynamics, molecular clock calibration and type. Time to the most recent common ancestor (in years), evolutionary rates (substitution/site/year) and marginal likelihood estimation results (estimated using Path Sampling (PS) and Stepping Stone (SS) methods) are reported.

Calibration	Clock	Population dynamics	tMRCA	Evolutionary rate	PS	SS
Inner Node Calibration	Strict	Constant	44037.89[95HPD:29026.41-58171.24]	$4.06\cdot10^{-6}$[95HPD:$2.08\cdot10^{-6}$-$5.54\cdot10^{-6}$]	-24209,147	-24209,314
		Skyline	44205.96[95HPD:30179.42-59729.17]	$4.03\cdot10^{-6}$[95HPD:$2.77\cdot10^{-6}$-$4.49\cdot10^{-6}$]	-24199,822	-24203522
	Relaxed LogNormal	Constant	84252.54[95HPD:38875.07-137000]	$2.911\cdot10^{-6}$[95HPD:$1.50\cdot10^{-6}$-$4.5\cdot10^{-6}$]	-23978,267	-23983,544
		Skyline	56615.81[95HPD:37288.85-76060.77]	$3.75\cdot10^{-6}$ [95HPD:$2.39\cdot10^{-6}$-$5.31\cdot10^{-6}$]	-23976,57	-23977,122
Tip date onlyu	Strict	Constant	187.12[95HPD:112.12-209.51]	$5.67\cdot10^{-3}$[95HPD:$1.88\cdot10^{-3}$-$3.21\cdot10^{-3}$]	-24208,941	-24208,276
		Skyline	212.14[95HPD:156.82-268.77]	$8.52\cdot10^{-4}$[95HPD:$6.33\cdot10^{-4}$-$1.08\cdot10^{-3}$]	-24184,036	-24186,849
	Relaxed LogNormal	Constant	150.11[95HPD:128.17-202.49]	$2.53\cdot10^{-3}$[95HPD:$1.39\cdot10^{-3}$-3.77^{-3}]	-23961,061	-23963,263
		Skyline	163.03[95HPD:118.58-215.45]	$1.21\cdot10^{-3}$[95HPD:$8.80\cdot10^{-4}$-$1.56\cdot10^{-3}$]	-23953,934	-23956,166

Table S2. Summary table of metadata available for the samples included in the present study.

SampleID	Municipality	Forest station	CanineCV	Sex	Age Category	Collection date
**FoxVa_1**	Brissogne	Nus	Neg.	Female	Adult	2009/2010
**FoxVa_2**	Charvensod	Aosta	Neg.	Female	Adult	2009/2010
**FoxVa_3**	Quart	Nus	Neg.	Male	Adult	2009/2010
**FoxVa_4**	Nus	Nus	Neg.	-	-	2009/2010
**FoxVa_5**	Saint-Nicolas	Arvier	Neg.	Female	Adult	2009/2010
**FoxVa_6**	Saint-Nicolas	Arvier	Neg.	Male	Adult	2009/2010
**FoxVa_7**	Torgnon	Antey-Saint-André	Neg.	Male	Adult	2009/2010
**FoxVa_8**	Doues	Valpelline	Neg.	Male	Adult	2009/2010
**FoxVa_9**	Cogne	Aymavilles	Neg.	Male	Adult	2009/2010
**FoxVa_10**	Courmayeur	Pré-Saint-Didier	Neg.	Female	Juvenile	2009/2010
**FoxVa_11**	Châtillon	Châtillon	Neg.	Male	Adult	2009/2010
**FoxVa_12**	Sarre	Aosta	Neg.	Female	Adult	2009/2010
**FoxVa_13**	Avise	Arvier	Neg.	Male	Adult	2009/2010
**FoxVa_14**	Saint-Christophe	Aosta	Neg.	Female	Adult	2009/2010
**FoxVa_15**	Quart	Nus	Neg.	Female	Adult	2009/2010
**FoxVa_16**	Sarre	Aosta	Neg.	Male	Adult	2009/2010

**FoxVa_17**	Torgnon	Antey-Saint-André	Neg.	-	-	2009/2010
**FoxVa_18**	Sarre	Aosta	Neg.	Female	Adult	2009/2010
**FoxVa_19**	Gignod	Etroubles	Neg.	Male	Adult	2009/2010
**FoxVa_20**	Gignod	Etroubles	Neg.	Female	Adult	2009/2010
**FoxVa_21**	Saint-Rhémy-En-Bosses	Etroubles	Neg.	Male	Adult	2009/2010
**FoxVa_22**	Morgex	Morgex	Neg.	Male	Adult	2009/2010
**FoxVa_23**	Pont-Saint-Martin	Pont-Saint Martin	Neg.	Male	Adult	2009/2010
**FoxVa_24**	Bionaz	Valpelline	Neg.	Female	Adult	2009/2010
**FoxVa_25**	Arvier	Arvier	Neg.	Female	Adult	2009/2010
**FoxVa_26**	Quart	Nus	Neg.	Male	Adult	2009/2010
**FoxVa_27**	Nus	Nus	Neg.	Male	Adult	2009/2010
**FoxVa_28**	Torgnon	Antey-Saint-André	Neg.	Female	Sub-adult	2009/2010
**FoxVa_29**	La Salle	Morgex	Neg.	Female	Juvenile	2009/2010
**FoxVa_30**	Bionaz	Valpelline	Neg.	Female	Adult	2009/2010
**FoxVa_31**	Quart	Nus	Neg.	Female	Adult	2009/2010
**FoxVa_32**	Antey-Saint-André	Antey-Saint-André	Neg.	Male	Adult	2009/2010
**FoxVa_33**	Courmayeur	Pré-Saint-Didier	Neg.	Female	Adult	2009/2010
**FoxVa_34**	Doues	Valpelline	Neg.	Male	Adult	2009/2010
**FoxVa_35**	Doues	Valpelline	Neg.	Male	Adult	2009/2010
**FoxVa_36**	Aosta	Aosta	Neg.	Male	Adult	2009/2010
**FoxVa_37**	Torgnon	Antey-Saint-André	Neg.	Female	Adult	2009/2010
**FoxVa_38**	Valtournenche	Antey-Saint-André	Neg.	Male	Adult	2009/2010
**FoxVa_39**	Charvensod	Aosta	Neg.	Male	Adult	2009/2010
**FoxVa_40**	Fenis	Nus	Neg.	Female	Juvenile	2009/2010
**FoxVa_41**	Saint-Denis	Chatillon	Neg.	Female	Adult	2009/2010
**FoxVa_42**	Challand-Saint-Victor	Verres	Neg.	Male	Adult	2009/2010
**FoxVa_43**	Valgrisenche	Arvier	Neg.	Male	Adult	2009/2010
**FoxVa_44**	Cogne	Aymavilles	Neg.	Male	Adult	2009/2010
**FoxVa_45**	Saint-Rhémy-En-Bosses	Etroubles	Neg.	Male	Adult	2009/2010
**FoxVa_46**	Quart	Nus	Neg.	Female	Juvenile	2009/2010
**FoxVa_47**	Ayas	Brusson	Neg.	Male	Adult	2009/2010
**FoxVa_48**	Saint-Pierre	Villeneuve	Neg.	Female	Adult	2009/2010
**FoxVa_49**	Quart	Nus	Neg.	Female	Adult	2009/2010
**FoxVa_50**	Avise	Arvier	Neg.	Female	Adult	2009/2010
**FoxVa_51**	Saint-Pierre	Villeneuve	Neg.	Male	Adult	2009/2010
**FoxVa_52**	Pré-Saint-Didier	Pré-Saint-Didier	Neg.	Male	Adult	2009/2010

**FoxVa_53**	Doues	Valpelline	Neg.	Female	Adult	2009/2010
**FoxVa_54**	Torgnon	Antey-Saint-André	Neg.	Male	Sub-adult	2009/2010
**FoxVa_55**	Torgnon	Antey-Saint-André	Neg.	Female	Adult	2009/2010
**FoxVa_56**	Chatillon	Chatillon	Neg.	Male	Adult	2009/2010
**FoxVa_57**	Chatillon	Chatillon	Neg.	Male	Adult	2009/2010
**FoxVa_58**	Nus	Nus	Neg.	Male	Adult	2009/2010
**FoxVa_59**	La Magdeleine	Antey-Saint-André	Neg.	Male	Adult	2009/2010
**FoxVa_60**	Brissogne	Nus	Neg.	Male	Adult	2009/2010
**FoxVa_61**	La Thuile	Pré-Saint-Didier	Pos.	Male	Adult	2009/2010
**FoxVa_62**	Cogne	Aymavilles	Neg.	Male	Adult	2009/2010
**FoxVa_63**	Saint-Rhémy-En-Bosses	Etroubles	Neg.	Female	Adult	2009/2010
**FoxVa_64**	Pollein	Aosta	Neg.	Male	Adult	2009/2010
**FoxVa_65**	Brissogne	Nus	Neg.	Male	Adult	2009/2010
**FoxVa_66**	Sarre	Aosta	Neg.	Male	Adult	2009/2010
**FoxVa_67**	Nus	Nus	Neg.	Male	Adult	2009/2010
**FoxVa_68**	Valtournenche	Antey-Saint-André	Neg.	Female	Adult	2009/2010
**FoxVa_69**	Aosta	Aosta	Neg.	Female	Adult	2009/2010
**FoxVa_70**	Saint Marcel	Nus	Neg.	Female	Adult	2009/2010
**FoxVe_1**	Cortina D'ampezzo	Pezie	Neg.	Male	Adult	15/09/2017
**FoxVe_2**	Lorenzago Di Cadore	Pezze Lorenzago Di Cadore	Neg.	Male	Adult	17/09/2017
**FoxVe_3**	Lorenzago Di Cadore	Pezze Lorenzago Di Cadore	Neg.	Female	Adult	17/09/2017
**FoxVe_4**	Longarone	Igne	Neg.	-	Juvenile	17/09/2017
**FoxVe_5**	Tambre	Pian Canton	Neg.	Male	-	24/09/2017
**FoxVe_6**	Tambre	Rispen	Neg.	Male	-	23/09/2017
**FoxVe_7**	Pieve D'alpago	Campedel	Neg.	-	-	20/09/2017
**FoxVe_8**	Tambre	Federa	Neg.	-	-	20/09/2017
**FoxVe_9**	Chies D'alpago	Comune Di Chies D'alpago	Neg.	Female	Adult	20/09/2017
**FoxVe_10**	Chies D'alpago	Irrighe	Neg.	Male	Adult	29/09/2017
**FoxVe_11**	Tambre	Col Indes	Neg.	Male	Adult	28/09/2017
**FoxVe_12**	Tambre	Pian Canton	Neg.	Female	Juvenile	29/09/2017
**FoxVe_13**	Pieve Di Cadore	Nonciadize	Neg.	Male	Adult	20/09/2017
**FoxVe_14**	Alpago	Foran	Neg.	Male	-	04/10/2017
**FoxVe_15**	Tambre	Ciprian	Neg.	Female	-	04/10/2017
**FoxVe_16**	Tambre	Pian Canton	Neg.	Female	-	04/10/2017
**FoxVe_17**	Tambre	Pianon	Neg.	Female	-	02/10/2017
**FoxVe_18**	Biban	Biban	Neg.	Male	-	02/10/2017

**FoxVe_19**	Ponte Nelle Alpi	Soccher	Neg.	Male	-	11/10/2017
**FoxVe_20**	Tambre	Sant'anna	Neg.	Male	-	15/10/2017
**FoxVe_21**	Tambre	Sant'anna	Neg.	Female	Juvenile	22/10/2017
**FoxVe_22**	Tambre	Col Indes	Neg.	Female	Juvenile	22/10/2017
**FoxVe_23**	Medei	Medei	Neg.	Male	Juvenile	28/10/2017
**FoxVe_24**	Pieve Di Cadore	Nonciarize	Neg.	Female	Adult	07/10/2017
**FoxVe_25**	Tambre	Pian D'ort	Neg.	Female	Adult	29/10/2017
**FoxVe_26**	Tambre	Ciprian	Neg.	Female	Adult	01/11/2017
**FoxVe_27**	Pieve Di Cadore	Col Pian	Neg.	-	Juvenile	18/11/2017
**FoxVe_28**	Belluno	Secchi	Neg.	Male	Adult	09/12/2017
**FoxVe_29**	Sedico	Suppiei	Neg.	Male	Adult	09/12/2017
**FoxVe_30**	Sedico	Peron	Neg.	Male	Adult	06/12/2017
**FoxVe_31**	Sedico	Suppiei	Neg.	Female	Adult	09/12/2017
**FoxVe_32**	Sedico	Suppiei	Neg.	Male	Adult	09/12/2017
**FoxVe_33**	Belluno	Fiume Piave	Neg.	Male	Adult	10/12/2017
**FoxVe_34**	Tambre	Malolt	Neg.	-	Juvenile	06/11/2017
**FoxVe_35**	Tambra	Pian Delle Lastre	Neg.	-	Juvenile	06/12/2017
**FoxVe_36**	Pieve Di Cadore	Nebbiu'	Neg.	Female	Adult	27/11/2017
**FoxVe_37**	Trichiana	S.Antonio Di Tortal	Neg.	Female	Adult	13/12/2017
**FoxVe_38**	Trichiana	S.Antonio Di Tortal	Neg.	-	Juvenile	13/12/2017
**FoxVe_39**	Sedico	Longano	Neg.	Male	Adult	14/12/2017
**FoxVe_40**	Sedico	Longano	Neg.	Male	Adult	10/12/2017
**FoxVe_41**	Croda Rossa Belluno	Croda Rossa Nevegal	Neg.	-	Juvenile	17/12/2017
**FoxVe_42**	Tambre	Col Indes	Neg.	Male	Adult	20/12/2017
**FoxVe_43**	Comelico Superiore Bl	Dosoleso	Neg.	Male	Adult	18/03/2017
**FoxVe_44**	Comelico Superiore Bl	Dosoleso	Pos.	Male	Adult	06/02/2017
**FoxVe_45**	Santa Giustina	Formegan	Neg.	-	Adult	28/01/2017
**FoxVe_46**	Pedavena	Pedavena	Neg.	-	-	14/04/2017
**FoxVe_47**	Ponte Nelle Alpi	Col Cugnan	Neg.	Female	Adult	29/04/2017
**FoxVe_48**	Feltre		Neg.	-	Juvenile	04/06/2017
**FoxVe_49**	Pescul	Selva Di Cadore	Neg.	Female	Adult	16/05/2017
**FoxVe_50**	Cornei	Alpago	Neg.	Female	Adult	02/12/2017
**FoxVe_51**	Punta	Val Di Zoldo	Neg.	Female	Adult	23/10/2017
**FoxVe_52**	Le Fosse	Peron	Neg.	Female	Adult	06/12/2017
**FoxVe_53**	Curva Del Cristo	Belluno	Neg.	Female	Adult	17/01/2018
**FoxVe_54**	Valzella		Neg.	Female	Sub-adult	31/01/2018

**FoxVe_55**	Alpago	Valde	Neg.	Female	Adult	21/01/2018
**FoxVe_56**	Tambre	Malolt	Neg.	Female	Adult	14/01/2018
**FoxVe_57**	Tambre	Franamento Valturca Na	Neg.	Female	Adult	21/01/2018
**FoxVe_58**	Alpago	Tomas	Neg.	Female	Adult	21/01/2018
**FoxVe_59**	Farra D'alpago	Tomas	Neg.	Female	Adult	21/01/2018
**FoxVe_60**	Belluno	Antore	Neg.	Male	Adult	25/01/2018
**FoxVe_61**	Tambre	Pian Grant	Neg.	Male	Juvenile	24/09/2018
**FoxVe_62**	Puos D'alpago	Romascienz	Neg.	Male	Adult	25/01/2018
**FoxVe_63**	Puos D'alpago	Malolt	Neg.	Male	Adult	28/01/2018
**FoxVe_64**	Puos D'alpago	Malolt	Neg.	Male	Adult	28/01/2018
**FoxVe_65**	Puos D'alpago	Malolt	Neg.	Female	Adult	28/01/2018
**FoxVe_66**	Colle Santa Lucia	Passo Giau	Pos.	Male	Adult	08/10/2018
**FoxVe_67**	Belluno	Visome	Pos.	Male	Adult	04/01/2018
**FoxVe_68**	Belluno	Visome	Pos.	Female	Adult	04/01/2018
**FoxVe_69**	Belluno	Visome	Neg.	Female	Adult	04/01/2018
**FoxVe_70**	Cesiomaggiore	Calliol	Neg.	Female	Adult	11/10/2018
**FoxVe_71**	Lentiai	San Gervasio	Neg.	Female	Juvenile	01/11/2018
**FoxVe_72**	Comelico Superiore	Selvapiana	Neg.	Male	Adult	21/10/2018
**FoxVe_73**	Tambre	Federa	Neg.	Male	Adult	17/11/2018
**FoxVe_74**	Tambre	Pianon	Neg.	Male	Adult	23/11/2018
**FoxVe_75**	Comelico Superiore	Pista Pies	Neg.	Male	Adult	30/01/2018
**FoxVe_76**	Domegge Di Cadore	Ranzenigo	Neg.	Male	Adult	17/11/2018
**FoxVe_77**	Lentiai	Salet Di Sotto	Neg.	Male	Adult	16/12/2018
**FoxVe_78**	Cencenighe Agordino	Malos	Neg.	Male	Adult	14/12/2018
**FoxVe_79**	Comelico Superiore	Pian Formaggio	Neg.	Male	Adult	12/12/2018
**FoxVe_80**	Tambre	Federa	Neg.	Male	Adult	15/12/2018
**FoxVe_81**	Alpago	Poiatte	Neg.	Female	Adult	31/01/2018
**FoxVe_82**	Puos D'alpago	Le Rive	Neg.	Female	Adult	09/02/2018
**FoxVe_83**	Puos D'alpago	Le Rive	Neg.	Female	Adult	31/01/2018
**FoxVe_84**	Cavessago	Cavessago	Neg.	Female	Adult	01/03/2018
**FoxVe_85**	Belluno	Cirvoi	Neg.	Male	Adult	30/12/2017
**FoxVe_86**	Farra D'alpago	Coste	Neg.	Male	Adult	31/12/2017
**FoxVe_87**	Tambre 32010	Sant'anna	Neg.	Female	Adult	31/12/2017
**FoxVe_88**	Tambre	Manteo	Neg.	Male	Adult	03/01/2018
**FoxVe_89**	San Pietro In Campo	San Pietro In Campo	Neg.	Male	Adult	30/12/2017
**FoxVe_90**	San Pietro Di Cadore	San Pietro Di Cadore	Neg.	Male	Adult	23/12/2017

**FoxVe_91**	San Pietro Di Cadore	Schiaron	Neg.	Male	Adult	23/12/2017
**FoxVe_92**	San Pietro Di Cadore	Via Pier Fortunato Calvi	Neg.	Male	Adult	20/09/2017
**FoxVe_93**	Cesiomaggore	Bordugo	Neg.	Male	Adult	20/09/2017
**FoxVe_94**	Livinallongo Del Col Di Lana	Varda	Neg.	Male	Adult	06/04/2018
**FoxVe_95**	Fonzaso	Ai Giaroni	Pos.	Male	Adult	14/01/2018
**FoxVe_96**	Tambre	Sant'anna	Neg.	Male	Adult	13/01/2018
**FoxVe_97**	Alpago	Spert	Neg.	Male	Adult	14/01/2018
**FoxVe_98**	Alpago	Spert	Neg.	Female	Adult	14/01/2018
**FoxVe_99**	Farra D'alpago	Poiatte	Neg.	Male	Adult	23/12/2017
**FoxVe_100**	Farra D'alpago	Coste	Neg.	Male	Adult	23/12/2017
**FoxVe_101**	Tomas	Farra D'alpago	Neg.	Male	Adult	07/12/2019
**FoxVe_102**	Poiatte	Alpago	Neg.	Female	Adult	07/12/2019
**FoxVe_103**	Spert	Alpago	Neg.	Female	Sub-adult	26/01/2020
**FoxVe_104**	Valmaor	Chies D'alpago	Neg.	Male	Adult	26/01/2020
**FoxVe_105**	San Daniele	Chies D'alpago	Neg.	Male	Adult	26/01/2020
**FoxVe_106**	Val Maor	Chies D'alpago	Neg.	Male	Adult	26/01/2020
**FoxVe_107**	Le Ronche	La Valle Agordina	Neg.	Female	Juvenile	29/05/2020
**FoxVe_108**	Rufane	Puos D'alpago	Neg.	Male	Sub-adult	26/01/2020
**FoxVe_109**	Poiatte	Alpago	Neg.	Female	Sub-adult	19/01/2020
**FoxVe_110**	Tomas	Farra D'alpago	Neg.	Female	Adult	12/01/2020
**FoxVe_111**	Prese	Alpago	Neg.	Male	Adult	12/01/2020
**FoxVe_112**	Col Indes	Tambre	Neg.	Female	Adult	05/01/2020
**FoxVe_113**	Foran	Alpago	Neg.	Male	Adult	05/01/2020
**FoxVe_114**	Pianture	Farra D'alpago	Neg.	Male	Adult	05/01/2020
**FoxVe_115**	Col Indes	Tambre	Neg.	Female	Adult	17/10/2020
