# Supplementary figures and images for: Canine Circovirus in Foxes from Northern Italy: Where Did It All Begin?
Source: Pathogens. 2021 Aug 9;10(8):1002. doi: 10.3390/pathogens10081002 (PMC8400258; doi:10.3390/pathogens10081002)

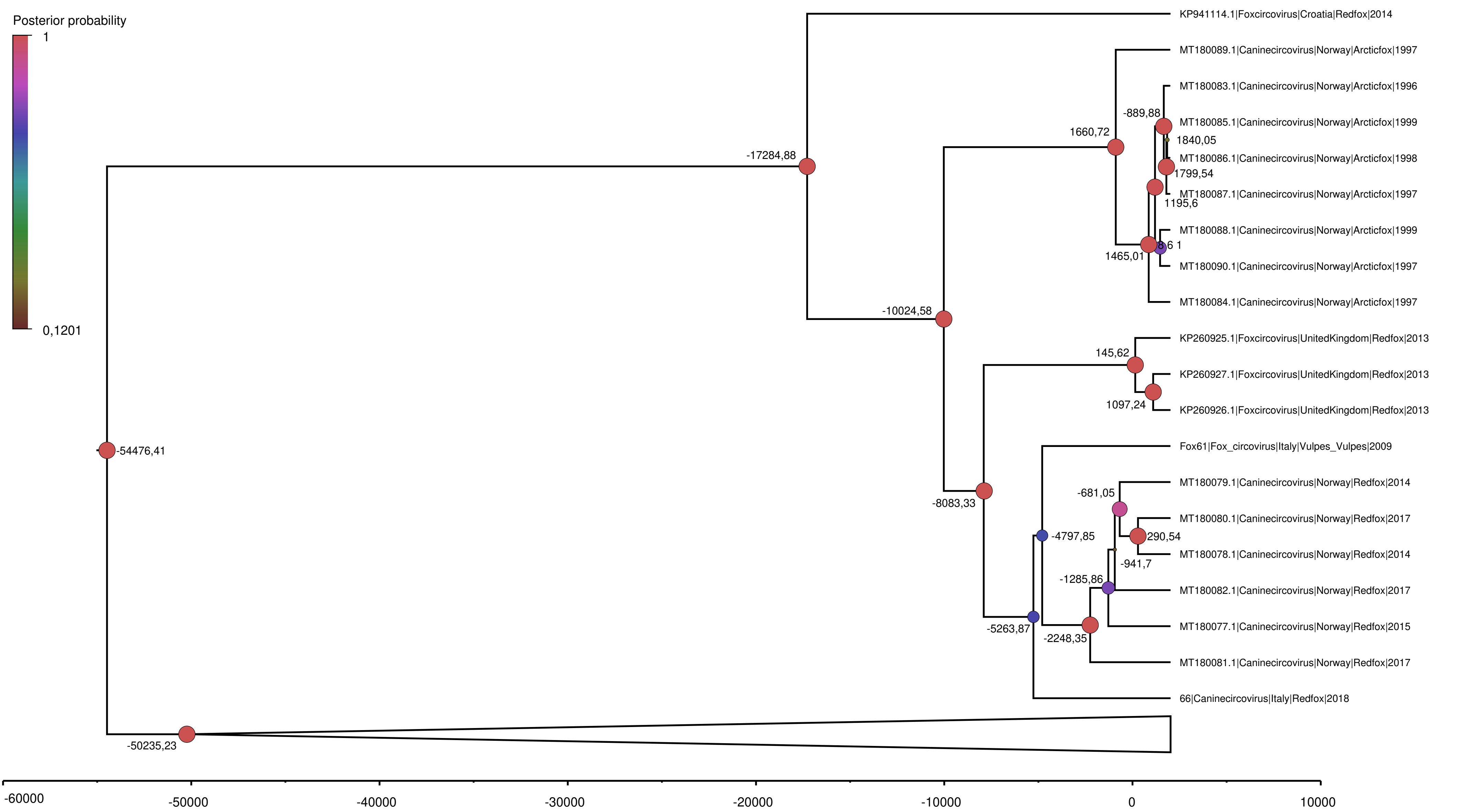

Supplement: Supplementary file 1 [file pathogens-10-01002-s001.zip › Figure S1.pdf]
